# Neuroprotective Effects of Celastrol in Neurodegenerative Diseases-Unscramble Its Major Mechanisms of Action and Targets

**DOI:** 10.14336/AD.2021.1115

**Published:** 2022-06-01

**Authors:** Dandan Liu, Qian Zhang, Piao Luo, Liwei Gu, Shengnan Shen, Huan Tang, Ying Zhang, Ming Lyu, Qiaoli Shi, Chuanbin Yang, Jigang Wang

**Affiliations:** ^1^Artemisinin research center, and Institute of Chinese Materia Medica, China Academy of Chinese Medical Sciences, Beijing, China.; ^2^Central People's Hospital of Zhanjiang, Zhanjiang, Guangdong, China.; ^3^Department of Geriatrics, Shenzhen People's Hospital, Shenzhen, China.; ^4^Department of Pharmacology, Yong Loo Lin School of Medicine, National University of Singapore, Singapore, Singapore.

**Keywords:** celastrol, neurodegenerative diseases, neuroprotective, quantitative chemical proteomics, target

## Abstract

There are rarely new therapeutic breakthroughs present for neurodegenerative diseases in the last decades. Thus, new effective drugs are urgently needed for millions of patients with neurodegenerative diseases. Celastrol, a pentacyclic triterpenoid compound, is one of the main active ingredients isolated from *Tripterygium wilfordii* Hook. f. that has multiple biological activities. Recently, amount evidence indicates that celastrol exerts neuroprotective effects and holds therapeutic potential to serve as a novel agent for neurodegenerative diseases. This review focuses on the therapeutic efficacy and major regulatory mechanisms of celastrol to rescue damaged neurons, restore normal cognitive and sensory motor functions in neurodegenerative diseases. Importantly, we highlight recent progress regarding identification of the drug targets of celastrol by using advanced quantitative chemical proteomics technology. Overall, this review provides novel insights into the pharmacological activities and therapeutic potential of celastrol for incurable neurodegenerative diseases.

Medicinal plant *Tripterygium wilfordii* Hook. f. (thunder god vine) based prescriptions have been widely used for inflammatory or autoimmune diseases in China clinical practice, including rheumatoid arthritis, nephritis, lupus erythematosus, thrombocytopenic purpura, etc [1]. Celastrol, one of the main bioactive substances present in *Tripterygium wilfordii* Hook. f., has a wide range of biological activities. Celastrol has been investigated as a potential therapeutic agent for various diseases, including cancer [2], neurodegenerative diseases [3] and obesity [4]. Neurodegenerative diseases are the most serious health problems in older adults worldwide, with the common characteristics of cognitive impairment, progressive degeneration, loss of neurons and myelin sheath in the central nervous system (CNS). With the aging community increasing, the number of patients with neurodegenerative disease will increase greatly and hunting for effective treatments or drugs with low side effects and clear efficacy is a matter of great urgency [5]. The potential superiorities of celastrol in CNS diseases have gradually become prominent. Many preclinical studies have confirmed that celastrol opens the possibility of therapy against multiple neurodegenerative diseases, including chronic neurodegenerative diseases such as Alzheimer’s disease (AD), Parkinson’s diseases (PD), Huntington’s disease (HD) and amyotrophic lateral sclerosis (ALS), and acute neurodegenerative diseases including stroke and traumatic brain injury (TBI) [3]. Here, we will comprehensively and critically introduce the major neuroprotective effects, mechanisms, and targets of celastrol in chronic and acute neurodegenerative diseases, and the potential side effects of celastrol will also be included. The detailed experimental studies of celastrol on CNS cells and neurodegenerative diseases in recent years are summarized in Table 1 and Figure 1.

**Table 1:** The detailed experimental studies of celastrol on CNS cells and neurodegenerative diseases.

Effects of celastrol on CNS cells and diseases	*In vitro* or *in vivo*	Models	Mechanisms of action of celastrol	Refs.
Microglia activation	Microglia cell line MG6	dsRNA [poly(I:C)]-induced activation	preventing F-actin rearrangement, preventing cytoskeletal alteration, attenuating the expression of proinflammatory cytokines and chemokines	10
	BV-2 microglia cells	LPS-stimulated activation	inhibiting LPS-induced phosphorylation of MAPK/ERK1/2 and NF-κB activation	11
	Female SD rats	SCI model	inhibiting the activation of microglia and microglia pyroptosis, down-regulating the release of pro-inflammatory cytokines and up-regulating the expression of anti-inflammatory cytokine and reducing the expression of NLRP3 inflammasome by inhibiting the expression of NF-κB/p-p65	12
	BV-2 microglia cells	LPS/ATP induced microgliosis		
Inflammatory responses of astrocytes	CRT-MG human astroglial cells	HIV-1 Tat (trans-activator of transcription)-induced inflammatory responses	inhibiting JNK, AP-1 and NF-κB activation and inducing expression of HO-1	16
		Poly (I:C) activated neuro-inflammation	suppressing ICAM-1/VCAM-1, chemokines expression and activation of JNK-STAT1 and NF-κB signaling pathways	17
Neuronal apoptosis and neuro-inflammation	PC12, SH-SY5Y cells and primary neurons	Cells were treated with Cd (10 μM and/or 20 μM) for 24 h	(1) inactivating JNK and Akt/mTOR signaling pathway and elevating PTEN activity; (2) inhibiting CaMKII-dependent Akt/mTOR pathway; (3) suppressing mitochondrial ROS-dependent AMPK-mTOR signaling pathway; (4) targeting NOX2-derived ROS-dependent PP5-JNK signaling pathway.	22-25
BBB dysfunction	Murine brain endothelial bEnd3 cells	OGD model	inducing activation of MAPKs and PI3K/Akt/mTOR pathways	30
AD	Male SD rats	i.p. injection of STZ, and inhale 3% sevoflurane for 2 h	All these dementias like pathology were reversed after celastrol treatment.	37
	Male SD rats	Aβ_25-35_-induced rat model of AD	Celastrol attenuated hippocampal inflammation, improved synaptic function, and maintained hippocampal energy metabolism.	38
	CHO cell line	A CHO cell line overexpressing Aβ	Celastrol inhibited Aβ_1-40_ and Aβ_1-42_ production by reducing the β-cleavage of APP, and reduced BACE-1 expression by preventing NF-κB activation.	18
	Transgenic mouse model of AD	A transgenic mouse model of AD overexpressing the human APP695sw mutation and the presenilin-1 mutation M146L (Tg PS1/APPsw)	Celastrol reduced the levels of Aβ, decreased the microgliosis in the cortex, and reduced the levels of both soluble and insoluble Aβ_1-38_, Aβ_1-40_ and Aβ_1-42_.	
	SH-SY5Y cells	Tau hyperphosphorylation induced by Aβ_1-42_	Aβ_1-42_ induced Tau hyperphosphorylation and HSP90 expression were inhibited by celastrol	39
	SH-SY5Y cells, C57BL/6J and APP23 mice primary hippocampal neurons	None	In addition to increased expression of HSP40, HSP70 and HSP90, celastrol induced activation of HSF1 and promoted the TTR transcription in SH-SY5Y cells.	40
	C57BL/6J, transgenic mouse model of AD	APP23 AD model mice, APP23/Ttr-/-(APP23 mice on Ttr knock-out background) mouse strains		
	H4 human neuroglioma cells stably transfected to overexpress human full length APP	LPS induced neuroinflammation	Celastrol increased HSP-70 and Bcl-2 expression, decreased NF-κB, COX-2, phosphorylated GSK-3β expression and ROS production.	43
PD	*Drosophila*	A *Drosophila* DJ-1A RNAi model of PD	Celastrol prevented the loss of DN and restored dopamine content to near normal levels.	45
	Swiss Webster mice	Dopaminergic neurotoxin MPTP-induced PD model	Celastrol attenuated the loss of dopaminergic neuron in the SNpc and reduced depletion of striatal dopamine levels, increased HSP70 expression to attenuate inflammation by preventing TNF-α and NF-κB activation.	19
	Male C57BL/6 mice and genetically modified mice (Nrf2-KO, NLRP3-KO and Caspase-1-KO)	MPTP-induced PD mouse model and AAV-mediated human α-synuclein overexpression PD model	Celastrol relieved motor deficits and nigrostriatal dopaminergic degeneration through Nrf2-NLRP3-caspase-1 pathway.	46
	SH-SY5Y cells	Rotenone-induced PD model	Celastrol suppressed oxidative stress, provided antiapoptotic effects to maintain the mitochondrial function and induced autophagy to clear damaged mitochondria.	47
	SH-SY5Y cells	SH-SY5Y cells were treated with 1 mM MPP^+^ for 24 h to induced about 50% neuronal death.	Celastrol treatment suppressed MPP^+^-induced injuries by activating autophagy through MAPK/p38, MAPK/ERK, MAPK/Akt, or MAPK/JNK signaling pathways.	26
	Male C57BL/6 mice	Mice received i.p. injections of MPTP (10 mg/kg/day for 3 days) 24 h after the last celastrol injection	Celastrol increased Bcl-2 expression in the substantia nigra by enhancing mitophagy to clear impaired mitochondria and further inhibiting dopaminergic neuronal apoptosis	
MS	Female C57BL/6 mice	EAE animal model	Celastrol suppressed pathogenic Th17 polarization in the peripheral lymph nodes, downregulated cytokine production in BMDCs and inhibited T cells infiltration into the CNS and Th17 cell responses in the CNS.	51
	Male SD rats	EAE animal model	Celastrol attenuated demyelination and inflammatory infiltration in spinal cord. Celastrol also attenuated optic neuritis by inhibiting cytokines and microgliosis production, expression of iNOS and activation of NF-κB in optic nerve, and attenuating ganglion cells apoptosis in the retina of EAE rats.	52
	Male C57BL/6 mice	EAE animal model	Celastrol modulated MAPK (p38, ERK) to regulate the downstream genes encoding SGK1, so as to restore the Th17/Treg balance and enhance BDNF expression in T cells, and lead to protection against EAE.	53
	Female SD rats	EAE animal model	Celastrol transformed cytokines profile from Th1 to Th2 pattern, with decreasing TNF-α and increasing IL-10 correspondingly. Celastrol also decreased NF-κB expression, nitrites levels, and immune-histochemical expression of TLR2 and CD3+ T-lymphocytic count.	54
ALS	Transgenic ALS mice	G93A transgenic familial ALS mice (high expresser line)	Celastrol inhibited proinflammatory cytokine and glial activation through reducing TNF-α, iNOS, CD40, GFAP and increasing HSP70 immunoreactivity in lumbar spinal cord neurons.	56
	Primary motoneuron cultures	Cellular stress, such as staurosporin and H_2_O_2_, to induce apoptosis and oxidative stress	Celastrol did not appear any neuroprotective effect and exhibited neurotoxic.	57, 58
Polyglutamine expansion diseases	HD Male Lewis rats	Succinate dehydrogenase inhibitor 3-NP-induced HD	Celastrol reduced neurotoxicity by decreasing the striatal lesion volumes, inducing HSP70 in the striata, and reducing astrogliosis.	19
	Polyglutamine aggregation and toxicity HeLa cells, PC12 cells, HSF1^+/+^ and HSF1^-/-^ mouse embryo fibroblast (MEF) cells	Polyglutamine aggregation and toxicity is transfection of a Q57-YFP fusion protein into cell lines	Celastrol effectively decreased the aggregation and toxicity of polyglutamine expression *in vitro* via stimulating HSF1 activity to lead to inducible HSP70 gene expression pathway.	60, 61
SCA14	SH-SY5Y, CHO, and COS-7 cells, primary cultured Purkinje cells	Adenovirus infection	Celastrol induced upregulation of HSP70 and HSP40 to synergistic diminish aggregation formation of mutant PKCγ and cells death. Celastrol activated autophagy also benefited for clearing the PKCγ aggregates.	63
	Male C57BL/6N mice	Pharmacological induction of HSPs	Celastrol treatment upregulated HSP70 by penetrating the mouse cerebellum.	
Stroke	Male SD rats	pMCAO model	Celastrol downregulated the expression of p-JNK, p-c-Jun and NF-κB.	67
	AIS patients	Clinical samples	Celastrol treatment increased IL-33 and IL-10 expression, and decreased IL-1β, IL-6, and TNF-α level *in vitro* and *in vivo*. The neuroprotective and anti-inflammatory effects of celastrol for ischemic stroke were derived from promoting growth ST2/IL-33 activation in microglia.	13
	Male SD rats	pMCAO model		
	Primary rats neurons and microglia	OGD model		
	Primary microglia-enriched cultures	Microglial polarization: microglia were transfected with a ST2 interference vector before pretreatment with OGD for 3 h, then treated with 50 ng/mL IL-33		
	Primary rats neuronal	Neurons underwent OGD for 3 h after which they were treated with different concentrations of IL-33		
	Male SD rats	Transient global cerebral ischemia reperfusion	Celastrol inhibited HMGB1/NF-κB signaling pathway.	69
	Primary rats neuronal	OGD model	Celastrol directly bound to HMGB1 to inactivate it, up-regulated HSP70 and down-regulated NF-κB expression to play neuroprotective effect in cerebral ischemia reperfusion injury *in vitro* and *in vivo*.	27
	Male SD rats	MCAO model		
	Male C57BL/6 mice	MCAO model	Celastrol exhibited neuroprotection and anti-apoptosis effects partially by modulating lipid metabolites.	70
	Hippocampal cell line (HT-22) cells	OGD model	Celastrol significantly attenuated I/R-induced hippocampal injury by inhibiting the AK005401/MAP3K12 signaling and activating the PI3K/Akt pathway.	71
	Male C57BL/6 mice	Bilateral common carotid clip reperfusion		
	Male SD rats	SAH endovascular perforation model	Celastrol attenuated brain swelling and protected BBB integrity after rats SAH by decreasing MMP-9 expression and attenuating pro-inflammatory cytokines expression.	31
TBI	*hsp110*-deficient mice, *hsp70.1* and *hsp70.3* (named *hsp70i*)-deficient mouse lines with C57BL/6 genetic background	Controlled Cortical Impact (CCI)	By increasing the levels of HSP70/HSP110, celastrol treatment in wild-type mice exhibited lower levels of brain injury, decreased cellular apoptosis, inflammatory cells infiltration and gliosis, and increased Ki-67-positive cells and improved behavior.	74

## Effects of celastrol on CNS cells

### Celastrol inhibits neuro-inflammation in microglia

Manipulation of the inflammatory responses offers an effective pathway for protecting brain cells from injuries induced by various neurodegenerative diseases. As the key innate immune cells in the CNS, rapidly activated microglial cells remove necrotic neurons in response to miscellaneous stress conditions in the nervous system. However, excessive activated microglial cells cause damages to the neurons, which may contribute to the pathogenesis of neuro-inflammatory disorders, such as neurodegenerative diseases [6] and autoimmune diseases of the CNS [7]. The activated microglia change ramified morphology to de-ramified and play a dual role in the CNS injuries according to the phenotypes activated. Chronic activated M1 phenotype is a detrimental state of microglia by producing neurotoxic substances to exacerbate brain injury, and transient M2 phenotype is a neuroprotective phenotype that followed by a shift to M1. Selective inhibiting M1 or increasing M2 phenotype polarization may be an effective strategy to decrease CNS inflammatory responses [8, 9]. It has been shown that celastrol skew M1 to M2 phenotype in microglia in several disease models. After microglial cell line MG6 activation, celastrol inhibited morphological changes of microglia by preventing F-actin rearrangement, prevented cytoskeletal alteration, attenuated the expression of pro-inflammatory cytokines and chemokines [10]. In lipopolysaccharide (LPS)-activated mouse microglia BV-2 cells, celastrol attenuated pro-inflammatory cytokines and nitric oxide (NO) production via inhibiting LPS-induced phosphorylation of MAPK/ERK1/2 and NF-κB activation [11]. In the rat spinal cord injury (SCI) model, celastrol inhibited the activation of M1 microglia in the spinal cord and microglia pyroptosis. Celastrol also down-regulated the release of pro-inflammatory cytokines, enhanced the expression of anti-inflammatory cytokines and reduced the expression of NLRP3 inflammasome by inhibiting the NF-κB/p-p65 expression [12]. In the rat permanent middle cerebral artery occlusion (MCAO) model, celastrol treatment reduced ischemia stroke-induced brain damage with reducing M1 expression and increasing M2 polarization [13]. These results give us a hint that celastrol inhibits neuro-inflammation in microglia by increasing M2-type to regulate the inflammation factors.

**Figure 1.**
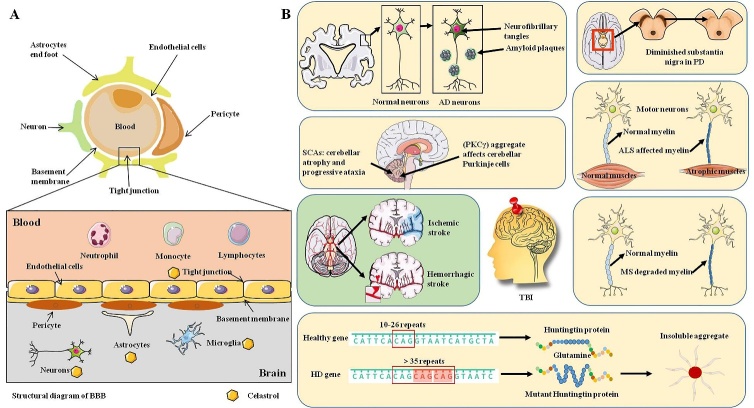
The detailed experimental studies of celastrol on CNS cells and neurodegenerative diseases. (A) The specific structure of BBB and the effects of celastrol on CNS cells. Celastrol inhibits neuroinflammation in microglia and astrocytes, prevents neuronal apoptosis and neuroinflammation, and maintains the integrity of BBB. (B) Preclinical studies on neurodegenerative diseases that have been studied with celastrol. Celastrol shows neuroprotective effects for multiple neurodegenerative diseases.

### Celastrol inhibits abnormal activated astrocytes

Astrocytes have significant effects in the CNS by maintaining neuronal homeostasis, regulating reactive oxygen species (ROS), preserving barrier functions for blood-brain barrier (BBB). Microglia and astrocytes usually become reactive in concert after pathological situations in the brain. Abnormally reactive astrocytes actively participate in the progression of neurodegenerative diseases [14, 15]. In HIV-1 Tat (trans-activator of transcription)-and poly (I:C) stimulated CRT-MG human astroglia cells, celastrol exerted anti-inflammatory effect by decreasing intercellular adhesion molecule-1 (ICAM-1) and vascular cell adhesion molecule-1 (VCAM-1) expression and inhibiting production of pro-inflammatory chemokines. These effects were mediated by preventing the signaling pathways of JNK MAPK-AP-1/NF-κB and inducing the expression of HO-1 [16, 17]. In the APP/PS1 AD mouse model, celastrol inhibited astrocytes activation and ameliorated AD pathologies [18]. In addition, celastrol powerfully reduced neurotoxin 3-nitropropionic acid (3-NP)-induced rat astrogliosis (reactive astrocytes) [19]. These results indicate that celastrol inhibits abnormal activation of astrocytes to reduce CNS inflammatory reactions.

### Celastrol prevents neuronal apoptosis and neuro-inflammation

The toxic transition metal Cadmiun (Cd) is currently one of the leading occupational and environmental exposure hazard factors mainly due to its water-soluble property. The long biological half-life (20-30 years in human) and low excretion rate of Cd responsible for its accumulation and toxicity in human organs. Cd possesses high BBB permeability, and induces neurotoxicity partly by increasing ROS, inflammation, neuronal apoptosis and finally may induce neurodegenerative diseases [20, 21]. In neurons, celastrol prevented Cd-induced neuronal apoptosis via the following signaling pathways: (1) inactivating JNK and Akt/mTOR signaling pathway and elevating PTEN activity [22]; (2) inhibiting CaMKII-dependent Akt/mTOR pathway [23]; (3) suppressing mitochondrial ROS-dependent AMPK-mTOR signaling pathway [24]; (4) targeting NOX2-derived ROS-dependent PP5-JNK signaling pathway [25]. Overall, celastrol has shown its unique advantages in preventing or reversing Cd-induced oxidative stress and neuronal apoptosis in neurodegenerative diseases. Celastrol also exerted neuroprotection in PD model by activating mitophagy to inhibit dopaminergic neurons (DN) apoptosis [26]. In addition, celastrol treatment significantly attenuated the DN loss in the substantia nigra pars compacta (SNpc) by inducing heat shock protein (HSP)70 expression and decreasing activation of inflammatory pathways [19]. In a recent study, we found that celastrol significantly reduced the primary rat cortical neurons damage caused by oxygen glucose deprivation (OGD) injury and decreased neurons pathological changes in male adult rats of MCAO model [27]. In general, celastrol prevents neuronal injury by inhibiting apoptosis and neuro-inflammation and increasing antioxidant stress capacity.

### Celastrol maintains the integrity of BBB

BBB acts as a goalkeeper by restricting access of blood-borne substances and cells to the CNS. CNS disorders often are accompanied by severe changes in the integrity and function of BBB by disrupting tight junction and changing the permeability. Restoring the tight junction is a promising target to maintain the CNS homeostasis and BBB integrity [28, 29]. In murine brain endothelial bEnd3 cells OGD model, celastrol could effectively maintain the tight junction integrity by ameliorating permeability of endothelial monolayers, attenuating loss of occludin, claudin-5 and zonula occludens-1 (ZO-1) as well as inhibiting macrophage migration [30]. These effects were related to celastrol induced activation of MAPKs and PI3K/Akt/mTOR pathways [30]. Celastrol ameliorated tight junction protein disruption after rat subarachnoid hemorrhage (SAH) to protect BBB integrity by decreasing MMP-9 expression and attenuating pro-inflammatory cytokines expression [31]. Celastrol is a BBB-penetrable compound, which may lay a foundation for maintaining the integrity of BBB and treating CNS disorders [32]. Overall, we have every reason to believe that celastrol could protect the damaged BBB to maintain the CNS homeostasis.

## Neuroprotective effects of celastrol in neurodegenerative diseases in pre-clinical studies

Neurodegenerative disorders are generally accompanied with slow consequent progressive dysfunction, as marked by dysregulation of neurons and axons in the CNS that affect different regions of the brain. Chronic neurodegenerative diseases mainly include AD, PD, ALS, HD, multiple sclerosis (MS) and spinocerebellar ataxias (SCAs), and acute neurodegenerative diseases mainly include stroke and TBI [33, 34]. A multitude of factors contribute to the pathogenesis of neurodegenerative diseases, including accumulation of unfolded/misfolded proteins, abnormal cellular transport, neuro-inflammation, oxidative stress, mitochondrial dysfunction, excite-toxicity, intracellular Ca^2+^ overload, abnormalities in the Genome, and apoptosis [35, 36].

### Celastrol and AD

As the most common form of dementia, two main neuropathological hallmarks exhibit in the brains of AD patients: (1) the extracellular neuritic plaques and insoluble Aβ species deposit; and (2) intracellular neurofibrillary tangles containing abnormal hyper-phosphorylated microtubule-associated protein Tau. Current treatments for AD in clinical mainly target symptomatic treatments with cholinesterase inhibitors and glutamate antagonists, and disease-modifying pharmacologic treatments are very limited [5]. Diabetes mellitus (DM) rats after sevoflurane anesthesia showed neuropathological changes similar to AD, including cognition impairment and reduced insulin-like growth factors-1(IGF-1) expression in the hippocampus. All these dementia-like pathologies were partially reversed after celastrol treatment [37]. Celastrol protected against Aβ_25-35_ induced rats learning and memory decline by attenuating hippocampal inflammation, improving synaptic function and maintain hippocampal energy metabolism [38]. Celastrol could effectively inhibit Aβ production in the CHO cells and AD mouse models, partly due to decreasing the rate-limited enzyme BACE-1 expression via preventing NF-κB activation [18]. In human neuroblastoma SH-SY5Y cells, Aβ_1-42_ induced Tau hyperphosphorylation and HSP90 expression were inhibited by celastrol [39]. Several groups showed that celastrol increased the expression of HSP40, HSP70 and HSP90 in SH-SY5Y cells. Celastrol also activated HSF1 to promote the transcription of systemic amyloid precursor transthyretin (TTR) in SH-SY5Y cells [40]. As an evolutionarily conserved protein, TTR serves as an endogenous detoxifier to bind Aβ peptides and regulate their aggregation in a chaperone-like manner in AD transgenic mouse model [41, 42], indicating that the role of celastrol in inhibiting Aβ aggregation may also contribute to its effects in alleviating AD pathology. In H4 human neuroglioma cell overexpressing human full-length APP (H4-APP), celastrol significantly reduced LPS-stimulated Aβ production and accumulation, and cells death. These neuroprotective effects of celastrol were derived from its anti-inflammatory and anti-oxidative stress effects by increasing Bcl-2 and HSP70 expression, decreasing NF-κB, COX-2, GSK-3β expression and ROS production [43]. These results suggest that celastrol has neuroprotective effects in preclinical AD models mainly by inhibiting Aβ production and accumulation through anti-inflammatory and anti-oxidative stress effects.

### Celastrol and PD

PD, the most common movement disorder, is mainly induced by progressive loss of DN in the SNpc, and the depletion of transmitter dopamine in striatum due to aggregation of misfolded α-synuclein, oxidative stress and mitochondria dysfunction [44]. Current pharmacological therapy of PD mainly aims at ameliorating motor symptoms by raising the concentration of functional dopamine in the striatum with dopamine receptor agonism or reducing dopamine metabolism, which could not alleviate or reverse the progression of PD [5]. In a *Drosophila* DJ-1A RNAi model of PD, with anti-inflammatory and antioxidant properties, celastrol prevented the loss of DN and restored dopamine content close to normal levels [45]. In MPTP-treated mouse model of PD, celastrol reduced neurotoxicity by attenuating the loss of DN in the SNpc and reducing depletion of striatal dopamine levels. Furthermore, celastrol increased HSP70 expression in the SNpc, and induced nuclear translocation of cytoplasmic HSP70 to generate newly expressed HSP70. Induced HSP70 expression attenuated inflammation by preventing TNF-α and NF-κB activation [19]. By using whole-genome deep sequencing analysis and multiple genetically modified mice, the authors verified that celastrol relieved PD deficits by targeting Nrf2-NLRP3-caspase-1 pathway [46]. In neurotoxin rotenone-induced PD model of SH-SY5Y cells, celastrol exhibited neuroprotective effect by suppressing oxidative stress, and showed anti-apoptotic effects to maintain the mitochondrial function and inducing autophagy to clear damaged mitochondria [47]. Autophagy plays a pivotal role in PD by removing damaged mitochondria and taking part in proteolytic degradation of α-synuclein [48]. Therefore, pharmacological enhancement of autophagy may be a viable strategy to combat α-synuclein aggregation and inhibit neuronal death in PD. Gene expression data (GSE8397) showed that autophagic activity in the substantia nigra of the midbrain in sporadic PD patients was lower than that of healthy controls [26]. In SH-SY5Y cells, celastrol treatment suppressed MPP^+^-induced mitochondrial membrane potential depolarization, ATP reduction and neuronal death by activating autophagy. In the PD mouse model, celastrol treatment significantly improved motor symptoms, decreased MPTP-induced dopaminergic neuronal death and increased Bcl-2 expression. By regulating autophagy and mitophagy-related genes, celastrol modulated the process of autophagosome biogenesis and raised mitophagy by degrading impaired mitochondria and further inhibiting dopaminergic neuronal apoptosis [26]. In general, celastrol exerts neuroprotective effects in PD models derived from multiple pharmacological properties, mainly including increasing HSP70 expression, activating autophagy, anti-inflammatory and antioxidant.

### Celastrol and MS

MS is a chronic immune-mediated neurodegenerative disease with unknown precise cause and pathogenesis. Inflammation, axonal loss, and demyelination are observed in accompany the very early phase of MS, which are affected by metabolic, oxidative stress, genetic and environment [49, 50]. Celastrol treatment significantly decreased clinical scores by delaying symptoms onset in experimental autoimmune encephalomyelitis (EAE) mice possibly via suppressing pathogenic T helper 17 (Th17) polarization in the peripheral lymph nodes, down-regulating cytokines production in bone-marrow derived dendritic cells (BMDCs), inhibiting T cells infiltration into the CNS and Th17 cells responses in the CNS [51]. Celastrol significantly alleviated neurologic severity of EAE rats by modulating the balance of cytokines to attenuate demyelination and inflammatory infiltration in spinal cord [52]. Acute inflammatory demyelinating optic neuritis may be an initial symptom of MS. Celastrol also attenuated optic neuritis by inhibiting cytokines and microgliosis production, expression of iNOS and activation of NF-κB in optic nerve, and attenuating ganglion cells apoptosis in the retina of EAE rats. Therefore, celastrol exhibited neuroprotective effects through T cells activities regulation, anti-inflammatory and anti-apoptotic effects [52]. In addition, celastrol inhibited the progression of EAE mice via restoring the Th17/Treg balance and enhancing brain-derived neurotrophic factor (BDNF) expression in T cells [53]. Celastrol also significantly transformed cytokines profile from Th1 to Th2 pattern, decreased TNF-α and increased IL-10 and correspondingly decreased NF-κB expression [54]. Based on the above findings, we conclude that celastrol may be a potential drug for MS treating in clinical.

### Celastrol and ALS

ALS is one of the most common rapidly progressive neurodegenerative disorders affecting the motor neurons and is clinically characterized by dysfunction of the upper and lower motor neurons and global muscle weakness [55]. In the G93A SOD1 transgenic mouse model of ALS, celastrol treatment delayed disease onset and increased survival rate by ameliorating weight loss, improving motor performance and rescuing lumbar spinal cord neurons loss. The neuroprotective effect of celastrol was related with the inhibition of pro-inflammatory cytokines and glial activation and the increase of HSP70 immuno-reactivity in lumbar spinal cord neurons [56]. The role of celastrol in the treatment of ALS is controversial at present. HSP70 activator arimoclomol protected the primary motoneurons from apoptosis and oxidative stress. While celastrol did not exhibit any neuroprotective effect under the same experimental conditions and exhibited neurotoxic and induced neuronal death even under unstressed condition. Therefore, the authors speculated that the neuroprotective effect of celastrol for the SOD1 mouse was due to its anti-inflammatory and antioxidant effects instead of its anti-apoptotic property [57, 58]. These results suggest that the neuroprotective mechanisms of celastrol in ALS may be debatable and complex, and more in-depth research is necessary in the future.

### Celastrol and HD

HD is an inherited autosomal dominant neuro-degenerative illness of the striatum, which is mainly triggered by excessive expanded polyglutamine segment near the amino terminus of huntingtin to form toxic aggregates [59]. The disorders of HD involve choreiform abnormal movements, cognitive deficits, and psychological changes. Currently, treatment of HD in clinical is only focus on symptoms alleviation [5]. After screening of 1040 FDA approved drugs and small bioactive compounds, celastrol was identified as a polyglutamine aggregation inhibitor [60]. Celastrol effectively decreased the aggregation and toxicity of polyglutamine expression *in vitro* via stimulating HSF1 activity to lead to HSP70 expression [61]. In neurotoxin 3-NP-induced rats HD model, celastrol reduced neurotoxicity by decreasing the striatal lesion volumes, inducing HSP70 expression in the striata, and reducing astrogliosis [19]. Overall, celastrol may be a therapeutic agent for treating polyglutamine expansion diseases, including HD.

### Celastrol and SCAs

The autosomal dominantly SCAs are a heterogeneous group of neurodegenerative disorders marked by progressive ataxia and cerebellar atrophy. Effective therapy or drug developments are hampered due to the heterogeneity of the SCAs. Genetically, SCAs include > 40 distinct subtypes based on different genetic loci of the causal gene [62]. More concretely, SCA14 has a frequency of 1-4% of families with SCA and is caused by a missense mutation or deletion of the PRKCG gene to induce neurotoxic amyloid-like fibril protein kinase Cγ (PKCγ) aggregate and affect cerebellar Purkinje cells [62]. *In vitro*, HSP90 inhibitor celastrol induced up-regulation of HSP70 and HSP40 to synergistic diminish aggregation formation of mutant PKCγ and cells death. Furthermore, celastrol activated autophagy also benefited for clearing the PKCγ aggregates. *In vivo*, celastrol treatment resulted in up-regulating of HSP70 expression by penetrating the mouse cerebellum. Therefore, HSPs were key endogenous regulators of pathophysiological PKCγ aggregation and HSP90 inhibitor celastrol may be a potential therapeutic medicinal component for treating SCA14 [63]. Celastrol may also play neuroprotective roles in other SCAs subtypes, and relevant research needs to be continued.

### Celastrol and stroke

Stroke can be divided into ischemia stroke and hemorrhagic stroke (including intracerebral and subarachnoid hemorrhage) and is one of the main causes of long-term disability and death worldwide. Acute ischemia stroke (AIS) is result from thrombosis or embolism occluding a cerebral vessel to induce sudden loss of blood supply to an area of the brain [64]. Recombinant tissue plasminogen activator (rt-PA) is the only pharmacological agent approved by FDA to selectively treat 1-8.5% of hospitalized AIS patients with short time window of 3-4.5 h after the onset of symptoms [65]. Celastrol might have therapy potential for ischemia diseases, for it promoted neovascularization in ischemia tissue by improving the mobilization, migration and tube formation capacities of transplanted endothelial progenitor cells (EPCs) in a mouse hindlimb ischemia model [66]. This result gave a hint that celastrol might increase EPCs therapy effect in stroke. In permanent cerebral ischemia model of rats, celastrol reduced brain water content, neurological deficit and infarct volume by down regulating the expression of p-JNK, NF-κB and p-c-Jun [67]. Celastrol treatment increased anti-inflammatory cytokines (IL-33 and IL-10) expression, and decreased inflammatory cytokines (IL-1β, IL-6, and TNF-α) levels *in vitro* and *in vivo* of permanent cerebral ischemia model [13]. The neuroprotective and anti-inflammatory effects of celastrol for ischemia stroke were derived from promoting growth stimulation expressed gene 2 (ST2)/IL-33 activation in microglia [13]. ST2 together with its ligand IL-33 stimulated M2 microglia to produce IL-10, thus improving neurons survival and mitigating cerebral injury after ischemia stroke [68]. In addition, celastrol exhibited anti-inflammatory and antioxidant effects by inhibiting high mobility group box 1 (HMGB1)/NF-κB signaling pathway in rats of transient global cerebral ischemia model [69]. In our recent study, we presented evidence that celastrol directly bound to HMGB1 to inactivate it, up-regulated HSP70 and down-regulated NF-κB expression to play a neuroprotective effect in cerebral ischemia-reperfusion (I/R) injury *in vitro* and *in vivo* [27]. Lipidomics and enrichment analysis after MCAO model in mice showed that celastrol exhibited neuroprotection and anti-apoptosis effects partially by modulating lipid metabolites in sphingolipid and glycerophospholipid metabolism pathways [70]. Celastrol significant attenuated I/R-induced hippocampal injury by inhibiting the AK005401/MAP3K12 signaling and activating the PI3K/Akt pathway [71]. In addition, celastrol attenuated brain swelling and protected the barrier function of BBB in the SAH rats [31]. Overall, celastrol shows obvious neuroprotective roles in a variety of stroke models, and how to promote its clinical transformation is the focus of next research.

### Celastrol and TBI

TBI caused by external force is among the most common causes of death and long-term disability in young adults with limited therapeutic options. Apart from the acute clinical manifestations, patients with a single severe TBI or repeated mild TBI also contribute to the risk of developing neurodegenerative symptoms in later life, such as psychiatric symptoms, personality change, early dementia and cognitive decline [72]. Considering oxidative stress mediates injury of neuro-inflammation and glutamatergic excite-toxicity, it is speculated that oxidative stress bridge TBI to subsequently increased risk of neurodegeneration via oxidation or carbonylation of key proteins [73]. Compared with wild-type mice, HSP110 or HSP70 deficient mice had increased brain injury and neurons death in response to TBI, which was partly due to the increased production of ROS, subsequently increased expression of pro-apoptotic gene p53 and p53-induced genes (Pigs). Celastrol or BGP-15 (increase the level of HSP70/HSP110) treatment in wild-type mice exhibited lower levels of brain injury, decreased cellular apoptosis, inflammatory cells infiltration and gliosis, and increased Ki-67-positive cells and improved behaviors [74]. These results suggest that it is necessary to research the pathophysiological mechanisms of TBI and neurodegenerative diseases, in order to identify more potential pathways that celastrol influenced in the TBI.

## Key directly binding targets of celastrol in neurodegenerative diseases

**Figure 2.**
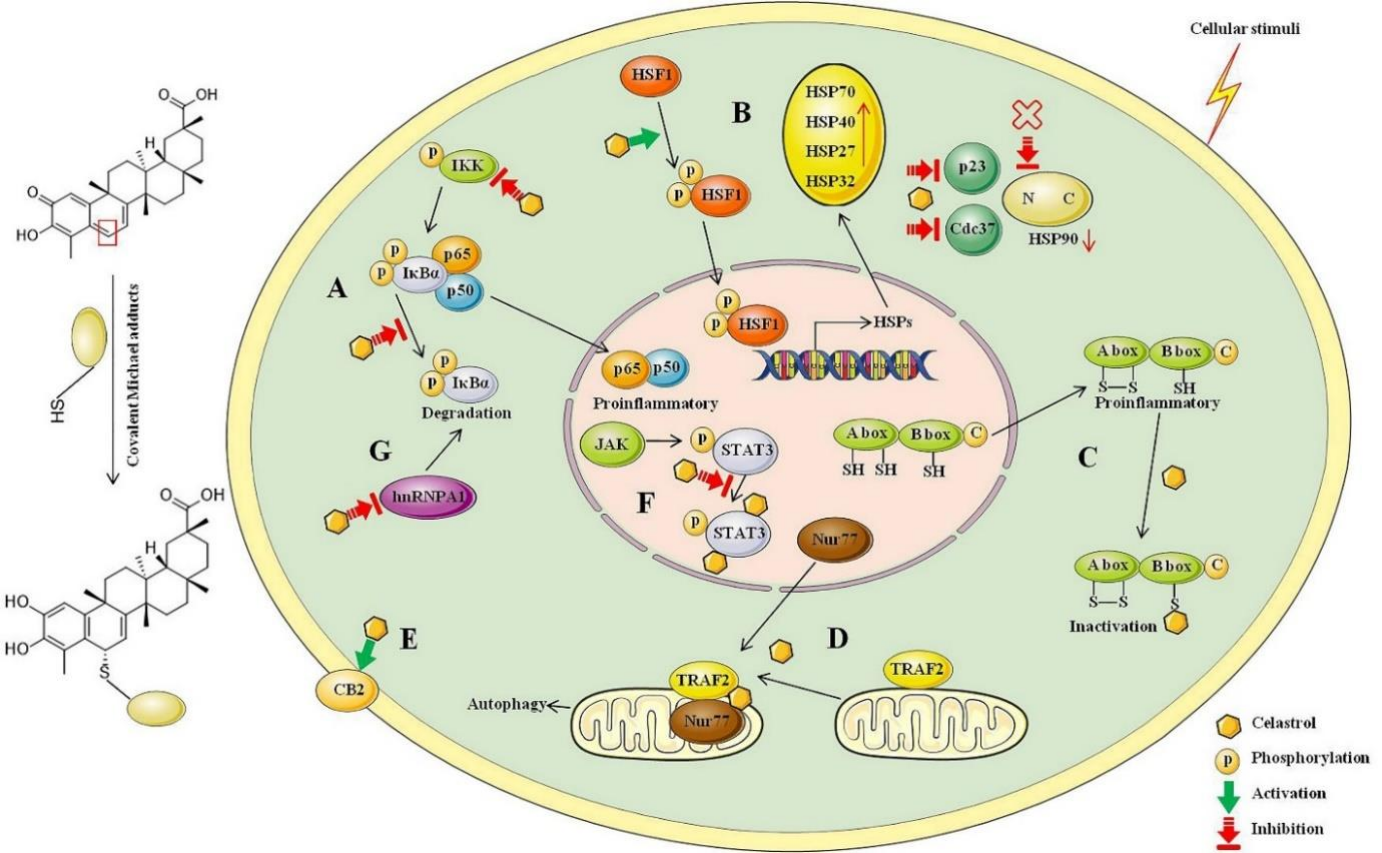
Proposed schematic diagram of celastrol forming covalent Michael adducts through the binding of electrophilic sites with the nucleophilic thiol groups of cysteine residues and directly binding targets. (A) Celastrol suppresses NF-κB activation by inhibiting IKKα and IKKβ activity and inhibiting IκBα phosphorylation to decrease degradation of IκBα. (B) Celastrol activates HSF1 to up regulate a wider set of potentially neuroprotective HSPs. Different from the existing N-terminal HSP90 inhibitor, celastrol covalently binds to HSP90 co-chaperone Cdc37 and p23 to disrupt the Cdc37-HSP90 or p23-HSP90 complex. (C) Celastrol directly binds to HMGB1 and inhibits the proinflammatory activity of disulfide isoform HMGB1. (D) Celastrol promotes mitochondrial ubiquitination and autophagy by covalently binding to Nur77 and inducing Nur77 interaction with TRAF2 to inhibit the classical IKK/NF-κB pathway. (E) Celastrol is a direct and selective CB2 agonist and triggers several CB2-mediated downstream signaling pathways to reduce inflammatory responses. (F) Celastrol directly binds and inhibits STAT3 tyrosine phosphorylation and nuclear translocation. (G) Celastrol accelerates the degradation of hnRNPA1 by directly binding with it and modulates hnRNPA1-IκBα-NF-κB-TNF-α pathway.

Celastrol interacts with numerous proteins and biological pathways to exert many physiological regulatory roles. Large-scale quantitative proteomics performed in human lymphoblastoid cells demonstrated that various biological pathways were regulated by celastrol, with 158 cellular core proteins were altered at least 1.5 fold in protein levels and 375 proteins as the “mitochondrial core proteins” [75]. Celastrol is a thiol reactivity compound. C2 of A-ring and C6 of B-ring in celastrol are prone to electrophilic reactions, which can undergo a regioselective Michael addition covalent product with the sulfhydryl group of cysteine residue [76]. Celastrol exhibited anti-inflammatory activity partly by suppressing TLR4 activation through inhibiting LPS binding to the TLR4/MD2 complex. The inhibitory effects of celastrol were derived from its thiol reactivity that allowed it covalently to bind to the cysteine to form Michael adducts [76]. Soft nucleophiles such as biologically relevant glutathione and cysteine add to the pharmacophore of celastrol in a remarkable stereospecific manner, and the stereo-specificity may explain its protein target selectivity [77]. The multiple biological effects of celastrol were blocked by incubation with excessive free thiols, which indicated a chemical mechanism for biological activity of celastrol via modification of key reactive thiols in proteins [78]. In addition, we have also found that celastrol covalently bound to active cysteine sites of peroxiredoxins (Prdxs) to induce cells apoptosis, thereby exerting anti-fibrotic effect, and the relevant data are being sorted out. Therefore, celastrol is a multi-target compound mainly influenced by its structure. The key directly binding targets of celastrol for neurodegenerative diseases, and the specific path diagrams are summarized in Figure 2.

### Celastrol is a NF-κB inhibitor

NF-κB is a family of nuclear transcription factors that implicate in diverse biological processes. The aberrant over-activated NF-κB is related with various diseases. NF-κB family has five different polypeptide subunits, including NF-κB1 (p50), NF-κB2 (p52), RelA (p65), RelB and c-Rel. The critical terminal components of the NF-κB signaling are NF-κB, IκB, and IκB kinase (IKK, consists of three subunits: IKKα, IKKβ and IKKγ also termed as NEMO). In a resting state, dimeric NF-κB is sequestered in the cytoplasm in a transcriptionally inactive state by binding to inhibitory IκBs proteins to form stable complexes [79]. Under a variety of cellular stimuli, activated IKK triggers site-specific phosphorylation of IκBα, then rapidly ubiquitinated and degraded by the proteasome complex. IκB degradation is necessary for NF-κB release and activation, and NF-κB translocates to the nucleus following IκBα degradation and activates the transcription of target genes [80]. As a NF-κB inhibitor, celastrol suppresses NF-κB activation and NF-κB target genes expression and has valuable potential for the intervention of NF-κB-dependent pathological conditions. Suppressing the NF-κB activation mainly relates with the following two mechanisms: (1) directly targeting cysteine 179 in the activation loop of IKKβ to inhibit IKKα and IKKβ activity; (2) restraining a variety of stimuli-induced degradation of IκBα through inhibiting IκBα phosphorylation [81]. Other proteins related to NF-κB may also be the targets of celastrol. For example, interleukin-1 receptor-associated kinases (IRAKs) have four different IRAK-like molecules: two active kinases (IRAK-1 and IRAK-4) and two inactive kinases (IRAK-2 and IRAK-M). All IRAKs take part in activation of NF-κB and interfering with small compound inhibitors at the level of IRAK-4 is the most immediate approach [82]. In the HepG2 cells treated with palmitic acid (PA) to activate TLR4/NF-κB, celastrol treatment effectively inhibited TLR4-dependent NF-κB activation and activated IRAKs. Knock-down IRAKs with small interfering RNA abolished PA-caused NF-κB activation, so targeting IRAKs was one way in which celastrol inhibited NF-κB activation [83]. As we mentioned earlier, celastrol exhibited neuroprotective effects by inhibiting HMGB1/NF-κB signaling pathway, down-regulated NF-κB expression in stroke and other diseases [27, 69]. These studies remind us that celastrol might be effective for many diseases related with NF-κB activation, and how to make good use of this binding mode to provide ideas for the development of celastrol or other drugs is an important research direction.

### Celastrol activates HSF1 and influences HSPs expression

HSF1 is an evolutionarily conserved transcription factor, which exists predominantly in an inactive form under normal conditions in cytoplasmic. In response to various stimuli, HSF1 translocates into the nucleus to induce the expression of genes encoding HSPs [84]. The impairment of HSF1 activity or its depletion exacerbates protein misfolding and aggregation, and decreases chaperone HSPs expression, thereby contributing to neurodegenerative diseases progression [84]. Under diverse cytoplasmic proteotoxic stimuli induced neurodegenerative disorders, small-molecule drugs that activate HSF1 could restore protein homeostasis [85]. Orally delivered celastrol promoted cells survival, inhibited inflammation, and maintained cellular homeostasis in apoptosis and inflammation animal disease models relying on HSF1-mediated protein homeostasis [86]. Under lethal stress in HeLa cells and SH-SY5Y cells, celastrol was cytoprotective by activating HSF1 with kinetics similar to heat stress. Celastrol activated HSF1 to induce the expression of chaperone genes (HSP70, HSP40, and HSP27), and lowered the temperature threshold required for the heat shock response [57]. Pharmacological activating HSF1 by celastrol treatment enhanced energy expenditure, mitochondrial function in fat and muscle to protect against obesity and metabolic dysfunction, which was achieved by activation of a peroxisome proliferator-activated receptor γ coactivator-1α (PGC-1α)-dependent metabolic program [87]. PGC1α is related with many pathological conditions associated with mitochondrial dysfunctions and impaired ROS balance, including aging and neurodegenerative disorders. We speculate that elastrol might play a central role in neurodegenerative diseases partly by activating HSF1-PGC1α transcriptional axis, and related research need to be implemented with considering precise control the induction levels of PGC1α for the treatment therapeutic window of PGC1α may be narrow [88].

Heat-inducible members have similar domain structures and functions, and function as molecular chaperones to mediate refolding or degradation of damaged intracellular proteins and enable cells to survive under harsh environments [89]. HSPs play a central role in initiating protein aggregates and removing aggregates to maintain protein homoeostasis, so targeting HSPs is a viable strategy to reduce pathogenic protein aggregates for developing neurodegenerative disorder drugs [89]. Celastrol upregulated a wider set of potentially neuroprotective HSPs including HSP27, HSP32 (also known as hemeoxygenase-1, HO-1), HSP70, and HSP70B [90, 91]. Celastrol-induced HSP70 localized to neuronal cells, HSP27 and HSP32 localized to glial cells in cerebral cortical cultures [92]. HSP90 stabilizes the client proteins by inhibiting their ubiquitination. In contrast, HSP70 along with its co-chaperone HSP40 promotes ubiquitination and proteasomal degradation. HSP90 and HSP70 possess opposed effects on client protein stability, so it is effective to put off diseases progression by targeting the protein quality control function of the HSP90/HSP70-based chaperone machinery to inhibit HSP90 or promote HSP70 function [93].

Be different from the existing classical HSP90 inhibitors such as geldanamycin, celastrol covalently bound to Cdc37 (binding site unknown) or formed either an intra- or intermolecular protein disulfide with Cdc37 to inactivate Cdc37 and disrupted the Cdc37-HSP90 complex [94]. Therefore, inducing the HSP90 client protein degradation and increasing HSP70 expression effects of celastrol were achieved by disrupting the protein-protein interaction of HSP90-Cdc37 instead of competing with ATP-binding pocket of HSP90 [95]. For the distinct HSP90 inhibiting mechanism with classic HSP90 inhibitors, celastrol reduced HSP90 interaction with the cochaperone p23 and showed mild synergistic effect with HSP90 inhibitors in anticancer research [96]. Classic inhibitors of HSP90 caused nonselective degradation of HSP90 client proteins. As the most sensitive target of celastrol, HSP90 co-chaperone p23 was bound by celastrol and co-chaperone structure was altered by altering its three-dimensional structure and triggering its oligomerization into amyloid-like fibrils, so as to more selective destabilization of steroid receptors compared with kinase clients [97]. Overall, celastrol maintains cellular homeostasis depending on HSF1-mediated protein homeostasis, up-regulating neuroprotective HSPs and down-regulating HSP90 expression.

### Celastrol directly binds to HMGB1 and inactivates it

HMGB1 is a ubiquitous nuclear protein and a key molecular target bound up with many distinct human diseases. Human HMGB1 consists of 215 amino acid residues and includes two homologous DNA-binding domains named HMGB1 A-box and B-box, and a negatively charged C-terminal acidic tail [98]. Three cysteines (Cys23 and 45 in the A box, and Cys106 in the B box) mainly determine the redox status of HMGB1. Only disulfide isoform HMGB1 with Cys23 and Cys45 form an intramolecular disulfide bond and Cys106 in reduced state possesses pro-inflammatory activity by binding to receptor TLR4 [98]. Extracellular HMGB1 secreted by passive or active release is an important mediator and a biomarker of multiple diseases. In various preclinical inflammatory related disease models, HMGB1 antagonists such as anti-HMGB1 mAb, metformin and glycyrrhetinic acid (GA), have been verified effective by decreasing HMGB1 expression, release and activity [99, 100]. Previous studies have shown that celastrol ameliorated inflammatory pain and played neuroprotective effect by suppressing the activated HMGB1/NF-κB signaling pathway, and relieved rats myocardial I/R injury by inhibiting HMGB1 expression [69, 101, 102]. In our recent study regarding the effects of celastrol on cerebral I/R, we discovered that celastrol directly bound to HMGB1 and did not affect the expression, secretion and redox states of HMGB1. Celastrol inhibited the proinflammatory activity of disulfide isoform HMGB1 and HMGB1 B box by binding to it and preventing the binding of HMGB1 to its inflammatory receptors TLR4 and RAGE [27]. Our results suggested that celastrol inactivated HMGB1 to exhibit anti-inflammatory effect in ischemia stroke or other diseases. The deficiency is that the specific binding sites of celastrol to HMGB1 protein is uncertain in our research, and leaves something to be desired.

### Celastrol is a ligand of Nur77 and covalently binds to Nur77

Nur77 (also known as TR3, NGFI-B and NR4A1), an orphan member of the nuclear receptor super family, acts as a promising target for anti-inflammatory therapy in metabolic and inflammatory diseases. Upregulation of Nur77 possesses neuroprotective effect and suppresses the pro-inflammatory genes expression through negatively regulating microglia activation-mediated dopaminergic neurotoxicity [103]. In astrocytes, increasing Nur77 expression with NR4A receptor ligand prevented nuclear export of Nur77, suppressed the transcriptional activity of NF-κB and its regulated inflammatory and apoptotic genes [104]. Nur77 was a direct intracellular anti-inflammatory target of celastrol by clearing inflamed mitochondria. Celastrol inhibited acute liver inflammation and chronic inflammation in obese animals by binding to Nur77 and promoting Nur77 translocation from the nucleus to mitochondria and interact with tumor necrosis factor receptor-associated factor 2 (TRAF2) [105]. TRAF2-Nur77 interaction inhibited TNF-α-induced ubiquitination of TRAF2 and Lys63-linked Nur77 ubiquitination. Ubiquitinated Nur77 interacted with p62/SQSTM1 by inducing autophagy to eliminate damaged mitochondria and contribute to anti-inflammatory function of celastrol. In addition, celastrol-induced Nur77 interaction with TRAF2 decreased the effects of TNF-α induced IκBα degradation by suppression of IKKα/β phosphorylation to inhibit the IKK-NF-κB pathway. In conclusion, celastrol alleviated inflammation through promoting mitochondrial ubiquitination and autophagy by inducing Nur77 interaction with TRAF2 and inhibiting the classical IKK/NF-κB pathway [105]. Six cysteines exist in Nur77 ligand-binding domain (LBD): C465, C475, C505, C534, C551 and C566. Celastrol covalently bound to the C551 reactive cysteine of Nur77 protein via a Michael addition reaction and induced Nur77 ubiquitination. C-6, one of the three electrophilic carbons in the quinone methide motif of celastrol, involved in an adduct formation. The covalent bond was reversible and required specific noncovalent interactions with Nur77 to position celastrol next to the thiol group of C551 [106]. Further research indicated that celastrol induced ubiquitination of Nur77 in the C-terminal and formed Nur77/p62 condensates coordinated multivalent interaction to mediate celastrol-induced mitophagy [107]. These results remind us think highly that cleastrol may also interact with Nur77 to exhibit neuroprotective effect in neurodegenerative diseases by inhibiting the IKK-NF-κB pathway.

### Celastrol is a CB2 agonist

Mounting evidence shows that cannabinoid receptors type 1 and 2 (CB1 and CB2) possess different physiological properties. CB1 agonists possess neuroprotective properties with psychoactive side effects at effective doses, and CB2 agonists mainly mediate anti-inflammatory, suppression of microglia activation, neuroprotective, and immunomodulatory actions in animal models of neurodegenerative diseases [108]. Based on split luciferase complementation assay (SLCA) screening method, celastrol was confirmed as a new direct and selective CB2 agonist. Celastrol triggered several CB2-mediated downstream signaling pathways, such as inhibiting cAMP accumulation, calcium mobilization, inducing CB2 receptor desensitization, CB2 medicated β-arrestin 2 trafficking and CB2-dependent ERK activation. By upregulating CB2 expression, celastrol reduced inflammatory responses, alleviated renal fibrosis, inflammatory and neuropathic pain and prevented the development of systemic sclerosis [109-111]. The selective CB2 activation effect of celastrol may lay the foundation for other neurodegenerative diseases [112].

### Celastrol inhibits STAT3 expression

By directly binding and inhibiting signal transducer and activator of transcription-3 (STAT3) tyrosine phosphorylation and nuclear translocation, celastrol protected against Ang II-induced cardiac dysfunction. Celastrol might bind to both SH2 (Gln-635/Val-637) domain and coiled-coil domain (CCD, Leu-207) of STAT3 to reduce its nuclear translocation and tyrosine phosphorylation [113]. In response to cytokines, hormones or growth factors, the canonical Janus kinases (JAK)-STAT3 pathway is activated through STAT3 phosphorylation, dimerization and accumulation in the nucleus. JAK-STAT3 pathway is emerging as a canonical inducer of astrocyte reactivity. STAT3 induces the astrogliosis reactivation, which plays key roles in modulating the adaptive responses in neurons and is a uniform response mechanism of the brain to acute or chronic neurological diseases [114]. Deleting STAT3 or chronic treatment with a systemic STAT3 inhibitor normalized cerebral network activity, attenuated neuroinflammation and ameliorated cognitive decline of AD model mice [115]. Therefore, celastrol may play a neuroprotective role partly by inhibiting STAT3 activation in various neurodegenerative diseases, and relevant research is scarce.

### Celastrol directly binds and down-regulates hnRNPA1 expression

Heterogeneous nuclear ribonucleoproteins (hnRNPs) are RNA-binding proteins that play complex biological processes. HnRNPs include 20 members in humans, and hnRNPA1 is the most abundant member of this family that regulates gene expression and RNA metabolism. Continuous evidence shows that hnRNPA1 plays an important role in the pathogenesis of neurodegenerative diseases, including familial ALS, MS, AD and stroke [116, 117]. By directly binding to the C terminus of IκBα, hnRNPA1 mediated the degradation of IκBα to influence the NF-κB activity and transcription of TNF-α [118, 119]. Our recent research also showed that celastrol accelerated the degradation of hnRNPA1 by directly binding with it, and modulated hnRNPA1-IκBα-NF-κB-TNF-α pathway to play a role in obesity-depression comorbidity [120]. Accordingly, it is speculated that celastrol may carry therapeutic potential for multiple neurodegenerative diseases by binding to hnRNPA1.

### By-effects caused by celastrol

**Figure 3.**
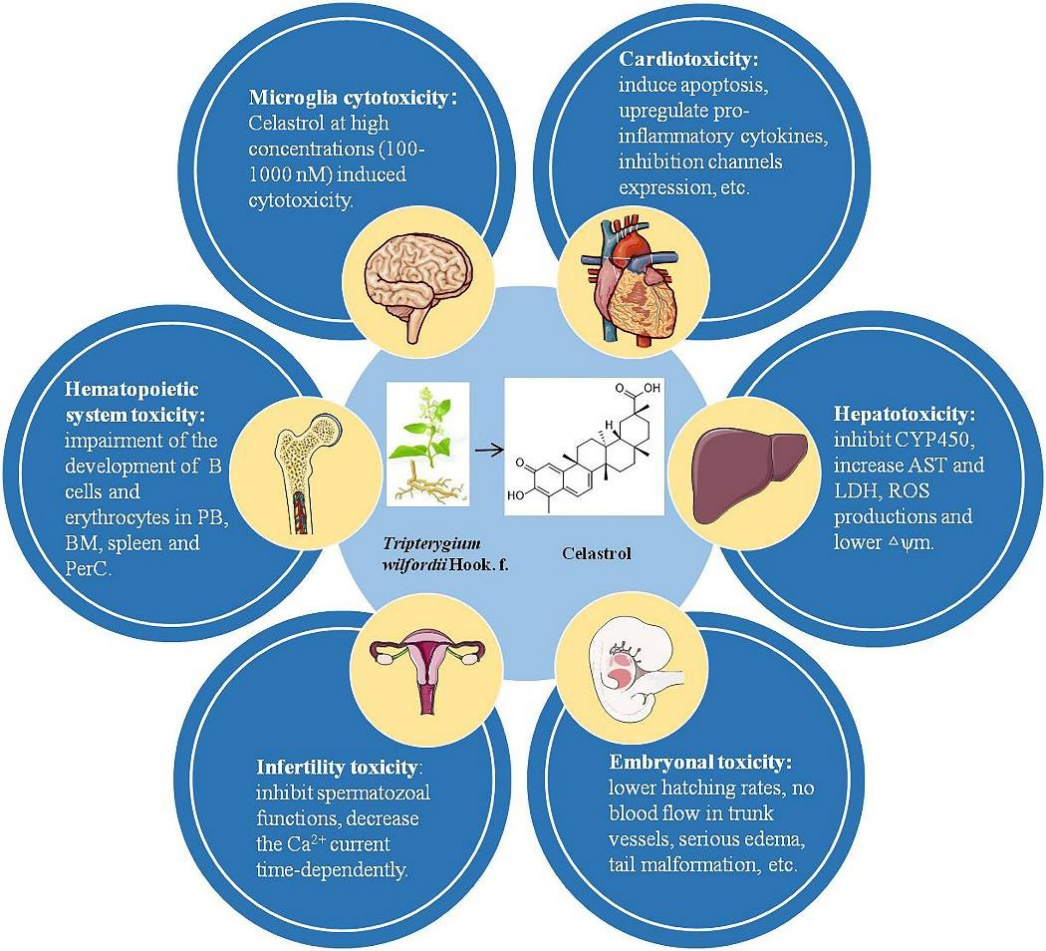
The main side effects of celastrol include microglia cytotoxicity, cardiotoxicity, hepatotoxicity, embryonal toxicity, infertility toxicity, hematopoietic system toxicity and potential risk of celastrol-drug interaction.

Due to the low water solubility, oral bioavailability, narrow dosage-window, short plasma half-life and side effects, clinical usage of celastrol is scarce at present. The major side effects of celastrol include infertility toxicity, cardiotoxicity, hepatotoxicity, hematopoietic system toxicity and so on [121]. The main toxicities of celastrol are presented in Figure 3. Next, we will introduce the studies in detail regarding the main side effects of celastrol.

### Microglia cytotoxicity

In the low concentration, celastrol reduced the inflammatory response of microglia. However, celastrol induced cytotoxicity in microglia at high concentrations (100-1000 nM), evidenced by lactate dehydrogenase (LDH) assay, reduced cells viability, and most of trypan blue-positive cells showed cytoplasm shrinkage [10]. As an HSP70 agonist, celastrol reduced postkindling seizure thresholds. Meanwhile, celastrol might also exert pro-inflammatory detrimental effects through enhancing microglia activation via HSP70 signaling [122].

### Cardiotoxicity

Celastrol showed cardiotoxic to zebrafish embryo with heart linearization, heart membrane hemorrhage, hemocytes accumulation in cardiac region and decreased heart rate [123]. Based on non-targeted metabolomics analysis, network toxicology study and biological approaches, male Wistar rats intraperitoneal injected with celastrol for successive 7 d (2 mg/kg/d, 1 mg/kg/d, 0.5 mg/kg/d) showed obvious heart damage, evidenced by significantly increased endogenous free fatty acid palmitic acid in plasma resulting in disrupted oxidative defense system. Excessive produced ROS further induced apoptosis by activating TNF signaling pathway and its downstream Caspase cascade [124]. Considering the druggability of celastrol as an anti-obesity medication, the security needs to be considered in the regulation of energy homeostasis. In an anti-obesity research, celastrol showed cardiovascular side effects with profound sympathoexcitatory effect, up regulating pro-inflammatory cytokines, potently reduced locomotor activity and potential muscle wasting [125]. Chronic exposure to celastrol potently inhibited both cardiac Kir2.1 and hERG potassium channels expression and caused QT prolongation to induce cardiotoxicity. Ascribe to the cardiotoxicity, celastrol acute blocked ion conduction, altered the rate of ion channel transport and reduced channel density on the cells surface [126].

### Hepatotoxicity

The cytochrome P450 (CYP) is an enzyme family of membrane-bound hemoproteins that takes part in the detoxification of xenobiotics, cellular metabolism and homeostasis. CYP enzymes inhibition lays the foundation for inducing drug-drug interactions. Celastrol concentration-dependently inhibited different isoforms of CYP450 in reconstructed human liver microsomes and might cause the herb-drug interactions [127]. In primary rat hepatocytes, as an inhibitor of CYP450 metabolic enzymes, celastrol treatment (3, 10 and 30 μM) induced obvious hepatotoxicity with increased AST, LDH, and ROS productions and lowered △ψm (low △ψm is a sign of early apoptosis). The celastrol hepatotoxicity was enhanced with 1-aminobenzotriazole (the broad-spectrum inhibitor of CYP450s) and decreased with phenobarbital (a CYP450s enzyme inducer) [128].

### Embryonal toxicity

Celastrol displayed toxicity to the normal development of zebrafish embryos in μM concentrations, confirmed by lower hatching rates, no blood flow in trunk vessels, serious edema in pericardial sac, tail malformation and lethal effect. No blood flow may also relate with the cardiotoxicity and antiangiogenic effects of celastrol [129]. However, whether celastrol exerts embryonal toxicity in rodents is unclear at present.

### Infertility toxicity

Celastrol dose-dependently inhibited guinea pig spermatozoal functions of forward motility, capacitation, the acrosome reaction, and sperm penetration assay. The acrosome reaction inhibitory effect was partly reversible when the exposure of spermatozoa to celastrol was less than 3 h [130]. Furthermore, in mouse spermatogenic cells and the progesterone-initiated sperm acrosome reaction, celastrol significantly decreased the Ca^2+^ current time-dependently and irreversibly to play antifertility effect [131].

### Hematopoietic system toxicity

For clarify the hematopoietic system effects of celastrol, mice were treated with celastrol daily intraperitoneal injection over the course of 4 days, and the 5 mg/kg/day celastrol treated mice showed signs of toxicity with hunched posture, ruffled fur and higher total white blood cells. Celastrol treatment showed hematotoxicity with specific impairment of the development of B cells and erythrocytes in peripheral blood (PB), bone marrow (BM), spleen and peritoneal cavity (PerC). In BM, the number of common lymphoid progenitors (CLP), common myeloid progenitors (CMP) and megakaryocyte-erythrocyte progenitors (MEPs) were also decreased, and the number of Granulocyte-Monocyte Progenitors (GMPs) increased. Therefore, celastrol may be a potential priming of CMPs to GMPs. Besides the hematotoxicity, based on the above results, proper dose of celastrol might be used to modulate the hematopoietic cells subset [132].

### Celastrol-drug interactions

UDP-Glucuronosyltransferases (UGTs), which share many similarities with CYP450, are glycoproteins localized in the endoplasmic reticulum (ER) and function as important phase II metabolic enzymes to detoxificate different endogenous and exogenous compounds. Inhibiting the activity of compounds to UGTs may affect clinical drug-drug or herb-drug interactions [133]. Apart from inhibiting CYP450 to give rise to the herb-drug interactions, celastrol potently inhibited the UGT1A6 and 2B7-mediated 4-MU glucuronidation reaction, prevented the UGT1A8 activity competitively and decreased the activity of UGT1A10 noncompetitively. Therefore, the risk possibility of celastrol-drug interactions or celastrol-containing herbs-drug interactions may also exist in this form [134, 135].

**Figure 4.**
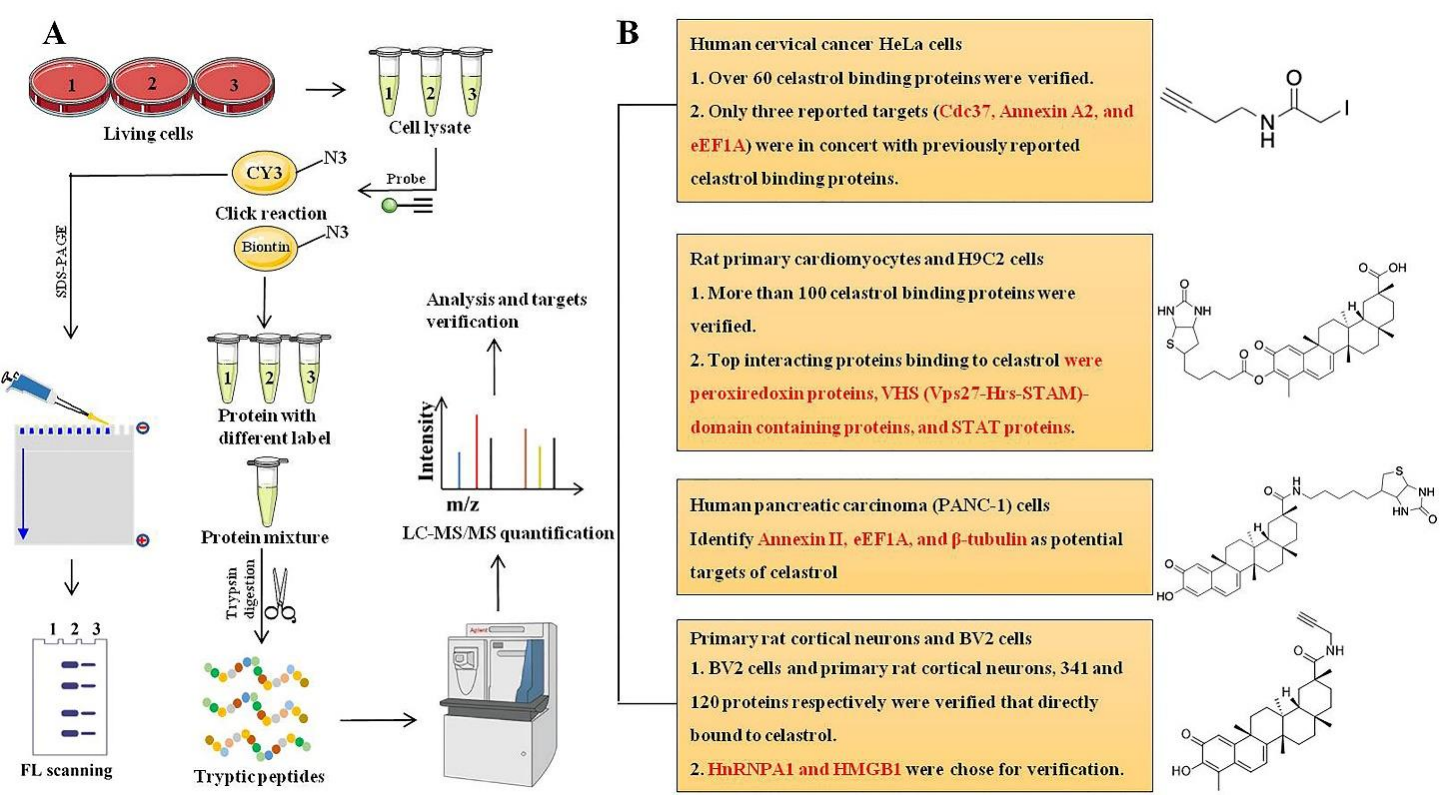
The small molecule compound probes available at present to research for celastrol targets, and specific flow chart of celastrol related quantitative chemical proteomics. (A) The specific flow chart of quantitative chemical proteomics. (B) Small molecule compound probes available at present to research for celastrol targets and verified targets in different kinds of cells.

Apart from the above findings, the dosage and/or treatment duration or certain experiment conditions may play a role for by-effects of celastrol. For example, celastrol displayed NADPH oxidase (NOX) enzymes inhibitory activity to exert antioxidant capacity, and treatment with celastrol led to an increased rate of H_2_O_2_ production and induced cells death at higher concentrations (≥ 50 μM) [136]. Though as a potent proteasome inhibitor to exert anticancer effect, celastrol did not show any neuroprotective effect under conditions of ubiquitin-proteasome system (UPS) inhibition in PD model. The toxic effects of celastrol in this PD model might result from its proteasome inhibiting effects, the cells type and culture conditions [137]. Based upon thiol related HSP90 inhibition effect, celastrol induced cells cycle arrest at G0/G1 phase in U937 cells. The target protein spectrum of celastrol became broader along with increase of dose. Similarly, side effects were also dose-dependent. The direct binding reactions and reversing effects of celastrol and thiol-containing agents may help mitigating side effects of celastrol. Thiol-containing agents decreased the targeting effect of celastrol on cellular thiols by forming competition with the cellular thiols [138]. In addition, protein denaturing or adding irreversible cysteine alkylation reagents also could partially disrupt the interaction of celastrol and protein thiols [139]. Overall, though celastrol exerts toxicity in multiple *in vitro* and *in vivo* models, future studies to further investigate the specific concentration range, time frame and underlying mechanism for the toxicity effects of celastrol are interesting topics. The studies on improving oral bioavailability, toxicity reduction and maximize efficacy of celastrol will further step into a new phase with the progress of nanotechnology, quantitative chemical proteomics, single cell sequencing and in-depth research of mechanisms. For example, nanotechnology-based numerous celastrol formulations have been widely investigated and showed great success to reduce systemic toxicity and enhance the bioavailability of celastrol [140, 141].

## Current available drug-probes for celastrol quantitative chemical proteomics research

Quantitative chemical proteomics has been applied to multifarious traditional medicine and small molecular compounds to identify small molecule-protein interactions, such as artemisinin, andrographolide, curcumin, aspirin, etc [142]. We summarize small molecule compound probes available at present to research for celastrol targets, and specific flow chart of celastrol related quantitative chemical proteomics is shown in Figure 4.

By utilizing an iodoacetamide-derived cysteine-reactive probe (iodoacetamide-alkyne, IA-yne), the authors identified over 60 binding proteins of celastrol in the human cervical cancer HeLa cells proteome via a competitive chemo-proteomics approach. However, only three reported targets (HSP90 co-chaperone Cdc37, Annexin A2, and elongation factor 1-alpha 1 (eEF1A)) were in concert with previously reported celastrol binding proteins, which may be due to the relatively low alkylation efficiency of celastrol to the rest proteins (HSP90β, Prostaglandin E synthase 3 (p23), NF-κB, Proteasomes, Peroxiredoxin-1, and β-Tubulin chain) [137]. Based on a biotin-labeled celastrol (Bio-celastrol), more than 100 proteins of celastrol potential targets were identified. Top interacting proteins binding to celastrol were peroxiredoxin proteins, VHS (Vps27-Hrs-STAM)-domain containing proteins, and STAT proteins [113]. Based on biotinylated celastrol-probes and performing affinity pull-down experiments in extracts of human pancreatic carcinoma (PANC-1) cells, the potential cellular targets of celastrol were identified (Annexin II, eEF1A, and β-tubulin). HSP90 appeared to be an indirect target of celastrol by invoking a redox imbalance [143]. Significantly, our research group developed an activity-based celastrol-probe (cel-p). Based upon this alkyne linked cel-p, we have studied the direct protein binding targets of celastrol in ischemia stroke and obesity-depression comorbidity. In BV2 cells and primary rat cortical neurons, we identified 341 and 120 proteins respectively, and we chose hnRNPA1 and HMGB1 proteins as the directly binding targets for verifying. [27, 120]. Furthermore, our team has also studied the treatment of liver fibrosis, sepsis and other CNS diseases with celastrol and cel-p (unpublished data).

## Conclusion

With the aging of population and changes in lifestyles, the number of patients suffering from neurodegenerative diseases is increasing, which represents an enormous burden in terms of both health and economic costs. A growing body of evidence suggests that the anti-oxidative stress and anti-inflammatory responses of celastrol significantly contribute to the pathology of these debilitating diseases. At present, the pharmacological and toxicological studies of celastrol are mainly concentrated in the preclinical stage, which possible due to used high concentrations and side effects. The above studies provide promising preclinical evidence of celastrol in the treatment of neurodegenerative diseases, which is of great value for further clinical study and evaluation. However, many therapies or small molecular compounds that prevent oxidative damage or inhibit neuroinflammation have exhibited good effect in preclinical studies, when converting from basic to clinical research there is little success. Deciphering the molecular codes of celastrol preserves promising prospects for medical science and clinical application to improve therapeutic strategies decrease side effects and extend a new departure. Furthermore, studies have carried out to confirm that celastrol has different effects at different doses, and how celastrol affects tissues at different doses still needs to be further clarified.
